# Impact of cryopreservation on DNA damage in *Acer platanoides* L. seeds evaluated by the comet assay

**DOI:** 10.1038/s41598-025-08476-3

**Published:** 2025-07-05

**Authors:** Beata P. Plitta-Michalak, Alice A. Ramos, Magdalena Trusiak, Ewelina Ratajczak, Marcin Michalak

**Affiliations:** 1 Department of Chemistry, Faculty of Agriculture and Forestry, University of Warmia and Mazury in Olsztyn, Plac Łódzki 4, Olsztyn, 10-719 Poland; 2 Institute of Biomedical Sciences Abel Salazar (ICBAS), University of Porto (U. Porto), Rua de Jorge Viterbo Ferreira 228, 4050-313 Porto, Porto, Portugal; 3 Interdisciplinary Center for Marine and Environmental Research (CIIMAR), University of Porto (U. Porto), Avenida General Norton de Matos s/n, Matosinhos, 4450-208 Portugal; 4 Department of Forestry and Forest Ecology, Faculty of Agriculture and Forestry, University of Warmia and Mazury in Olsztyn, Plac Łódzki 2, Olsztyn, 10-719 Poland; 5 Institute of Dendrology, Polish Academy of Sciences, Parkowa 5, Kórnik, 62-035 Poland

**Keywords:** Comet assay, Cryopreservation, DNA damage, 5-hydroxymethylcytosine, 5-methylcytosine, 8-oxo-7,8-dihydroguanine, Plant sciences, Plant biotechnology, Plant breeding, Plant stress responses, Biochemistry, DNA, Biotechnology, Plant biotechnology, DNA damage and repair, DNA methylation

## Abstract

Plant cells can suffer significant damage during cryopreservation, primarily due to extensive ice punctures and oxidative stress from reactive oxygen species (ROS). The extent of damage is closely linked to the moisture content (MC) of seeds, a crucial factor influencing their viability after cryostorage. However, there is limited information on the direct effects of MC in cryostored seeds on their overall (epi)genetic integrity. Maintaining genome integrity is essential for plant growth and ensures reliable transmission of genetic information. To investigate seed responses to genotoxic stress from desiccation and cryostorage, specific DNA damage, including DNA strand breaks, alkali-labile sites, and 8-oxo-7,8-dihydroguanine (8-oxoG), was measured, along with changes in epigenetic marks 5hmC (5-hydroxymethylcytosine) and 5mC (5-methylcytosine). This is the first study to demonstrate fine DNA strand breaks in orthodox seeds of *Acer platanoides* L. at varying MC immediately after cryostorage. Notably, 8-oxoG was detected for the first time in plant samples after cryostorage, and it was negatively correlated with seed viability, whereas the epigenetic marks showed a positive correlation. These findings indicate that cryostorage at non-optimal MC levels is genotoxic for seeds. Furthermore, the study demonstrated that the comet assay is a valuable technique for monitoring global DNA integrity in cryopreserved plant material.

**Supplementary Information:**

The online version contains supplementary material available at 10.1038/s41598-025-08476-3.

## Introduction

Global biodiversity faces a significant threat from climate change, deforestation, and human land use for food production, which underscores the critical importance of plant species preservation^1^. *Ex situ* seed conservation in seed banks is one of the approaches to addressing this challenge. However, the effectiveness of this method depends on factors such as species and storage conditions^2,3^. Traditional seed banking requires that seeds be stored in dry conditions at − 18 ± 3 °C. However, this is only suitable for desiccation-tolerant (orthodox) seeds. In contrast, recalcitrant and intermediate seeds cannot tolerate these conditions^3,4^, which suggests that approximately half of all plant species will not be suitable for conventional seed banking^4–7^. Consequently, cryobiotechnologies (cryogenic storage and in vitro technologies) are often required for effective long-term conservation of these species^6,8^. Nevertheless, cryopreservation techniques can be successfully applied to orthodox and intermediate seeds as a safety backup when the optimal moisture content (MC) is carefully determined^9–13^.

Chemical and physical damage to macromolecules, cells, and tissues can occur throughout the cryopreservation process, including cryoprotection, desiccation, cryostorage, and in vitro regeneration^14^. Genetic damage is a serious concern, and the (epi)genetic integrity of cryopreserved tissues and organs has been assessed in a number of species^15–19^. Genetic damage can result from mechanical damage caused by the crystallisation of intracellular and extracellular water at freezing temperatures, and from oxidative damage caused by the uncontrolled generation of reactive oxygen species (ROS) and insufficient antioxidant capacity^14,15,20,21^. Indeed, overproduction of ROS is toxic, causes damage to nucleic acids, proteins, and lipids, and leads to membrane oxidation^20–22^.

Understanding the genotoxic effects of cryopreservation techniques is a crucial step toward developing appropriate and effective methods for long-term *ex situ* conservation of plant material. DNA damage can be measured with the comet assay, a simple, sensitive, and versatile electrophoretic technique that detects various types of damage to nuclear DNA^23,24^. The alkaline version of the assay is most widely used to detect DNA strand breaks and alkali-labile sites (ALS). Modifications to the comet assay, including the digestion of nucleoids with lesion-specific endonuclease, have facilitated the detection of specific DNA nucleobase oxidation. Oxidized purines, such as 8-oxoguanine (8-oxoG) and 2,6-diamino-4-hydroxy-5-formamidopyrimidine (FaPyG), are identified with formamidopyrimidine-DNA glycosylase (FPG). This enzyme possesses AP-lyase activity and excises damaged bases to generate abasic sites that are subsequently transformed into strand breaks^25^, which are quantified using the comet assay.

The comet assay is widely used in animal science to analyze the impact of cryopreservation procedures on blood cells, saliva leucocytes^26–28^and sperm samples from wild and domesticated animals^29-32^as well as humans, as a complementary tool for diagnosing male reproductive health and infertility^33,34^. While this technique is valued in animal cryopreservation research, its applicability for assessing the impact of cryopreservation procedures on plant tissues has not been studied to date^35^. In plants, the comet assay has been used to evaluate DNA damage and repair in seed radicles at the protrusion stage during germination, and in studies of dry or imbibed seeds exposed to ionizing radiation or chemical treatments^36-40^. Recent research has shown that the comet assay is an effective and sensitive tool for monitoring fine changes in DNA integrity and the DNA repair process in recalcitrant plant germplasm subjected to gradual desiccation and accelerated aging^41,42^.

Since cryopreservation is currently used for long-term preservation of orthodox seeds, short-lived seeds, and endangered or economically valuable species, including tropical forest tree species^40–45^understanding the fundamentals of seed viability and resistance to genotoxic factors is crucial for the effective conservation of plant germplasm in seed banks. It is, therefore, necessary to test whether this storage method causes (epi)genetic changes.

Moisture content is one of the critical determinants of cryopreservation success. Thus, desiccation is often employed as a preliminary step in cryopreservation protocols to minimize the damaging effects of ice nucleation and to achieve the cooling rates necessary to induce vitrification of cytoplasm by increasing the viscosity of the aqueous solution within cells. Most studies indicate that desiccation to approximately 10% MC, at which the cytoplasm undergoes vitrification, increases the germination rate of orthodox seeds after cryostorage^43^. Consequently, this study comparatively analyzed changes in DNA strand breaks and epigenetic marks: 5-methylcytosine (5mC) and 5-hydroxymethylcytosine (5hmC), the accumulation of oxidized guanine (8-oxoG), and levels of reactive oxygen species (ROS) in relation to moisture content (MC) and the viability of *Acer platanoides* L. seeds. Orthodox seeds, which are resistant to desiccation, serve as an excellent model for distinguishing between the cellular damage resulting from water withdrawal and cryostorage.

As previously noted, 5mC plays a role in several essential biological processes, including heterochromatin formation, genomic imprinting, regulation of endogenous gene expression, defense against transposons, and the silencing of transgenes^44^. Additionally, global 5mC levels can indicate the viability of seeds with varying post-harvest physiology, as a decrease in m5C levels has been observed in seeds with diminishing germinability ^10,44,45^. In contrast, in highly viable seeds, 5mC levels remained constant or increased, which may be considered an adaptive mechanism to environmental stress and water withdrawal^41,46^. Therefore, to assess the epigenetic stability of seeds, the levels of m5C and its oxidized derivative, hm5C, were examined. Although hm5C did not appear to be a direct marker of seed viability, and its origin in plant genomes is debatable, it may impact genome stability and gene expression^44^.

## Results

### Changes in germination, seedling emergence, explant regeneration potential, respiratory activity, and membrane damage in seeds with different moisture content before and after cryostorage

**Fig. 1:**
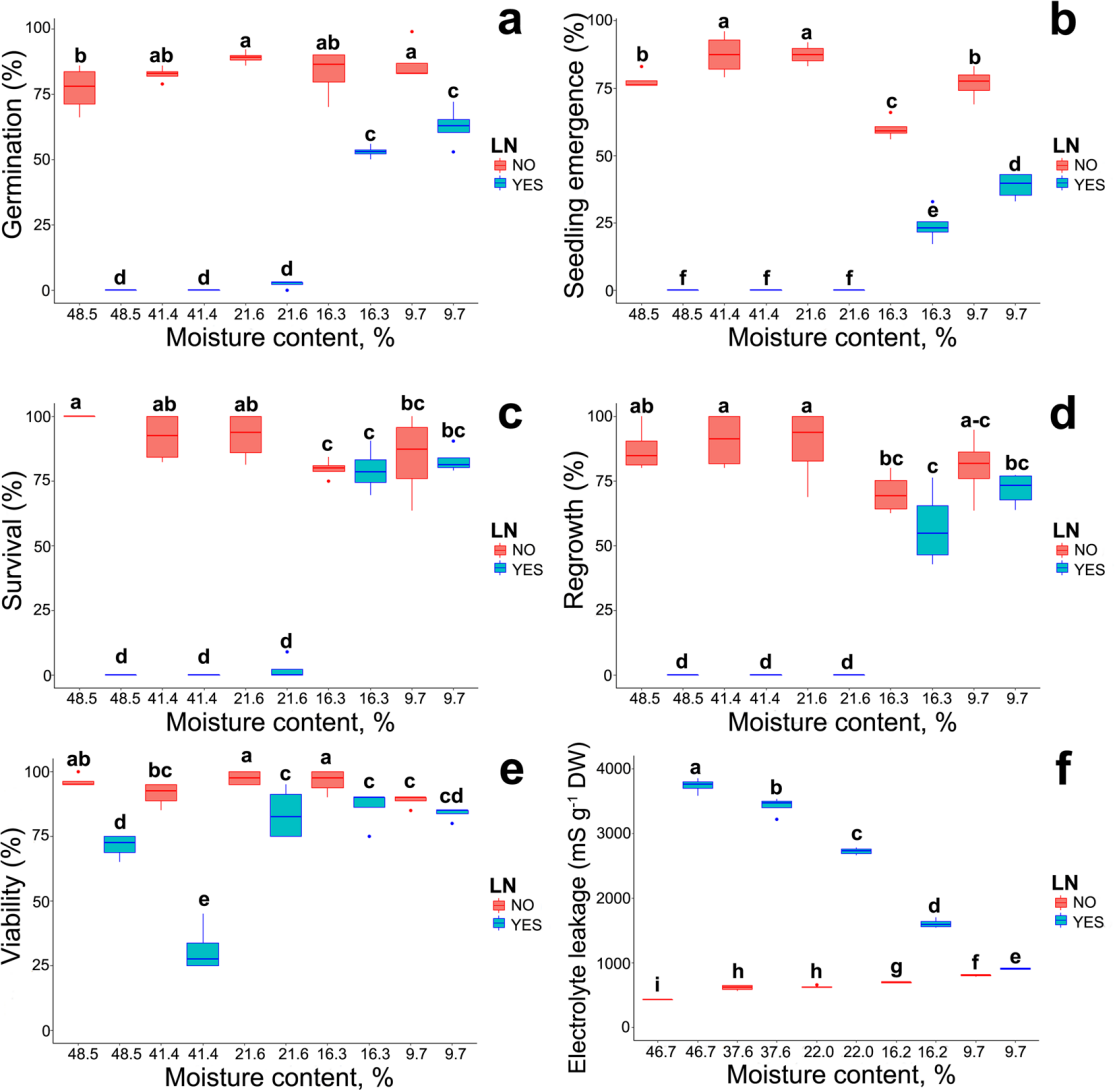
Effect of storage at different levels of MC in LN for 3 months on germination (a), seedling emergence (b), in vitro survival (c), in vitro regrowth (d), viability measured by TTC (e), and electrolyte leakage (f) of *Acer platanoides* L. seeds. Statistical analysis was conducted using GLMZ and Duncan test for germination (a), seedling emergence (b), in vitro survival (c), in vitro regrowth (d), viability measured by TTC (e), and non-parametric Kruskal–Wallis test was conducted for electrolyte leakage (f). Values marked with the same letter are not significantly different at *p* < 0.05. Lines denote the median, boxes the 25th to 75th percentile, while whiskers are the 5th to 95th percentiles.

The germination capacity of non-cryostored seeds ranged from 77 to 89%. In contrast, seeds stored in liquid nitrogen (LN) exhibited significantly lower germination capacity, which was highest (62.8%) in seeds with the lowest MC of 9.7%. Seeds with a higher MC (21.6–41.4%) did not germinate after storage in LN (Table 1; Fig. 1a).

**Table 1:** Moisture content (MC; %) (values in first line) and water content (WC; g^●^g^-1^) (values in the second line) of samaras, seeds, cotyledons, and embryonic axes (EA) of *Acer platanoides* L. after collection and desiccation.

	Samaras	Seeds	Cotyledons	Embryonic axes
Freshly collected Seeds (D1)	46.7 ± 0.01	48.5 ± 0.01	48.1 ± 0.01	52.6 ± 0.01
	0.88 ± 0.01	0.94 ± 0.04	0.93 ± 0.04	1.11 ± 0.05
First level of Desiccation (D2)	37.6 ± 0.01	41.4 ± 0.1	40.9 ± 0.01	45.5 ± 0.01
	0.60 ± 0.01	0.71 ± 0.02	0.69 ± 0.02	0.84 ± 0.03
The second level of Desiccation (D3)	22.0 ± 0.01	21.6 ± 0.01	22.8 ± 0.01	23.3 ± 0.01
	0.28 ± 0.01	0.28 ± 0.01	0.30 ± 0.01	0.3 ± 0.01
The third level of Desiccation (D4)	16.2 ± 0.01	16.3 ± 0.01	16.1 ± 0.01	15.3 ± 0.01
	0.19 ± 0.01	0.19 ± 0.01	0.19 ± 0.01	0.18 ± 0.01
The fourth level of Desiccation (D5)	9.7 ± 0.01	9.7 ± 0.01	9.8 ± 0.01	8.1 ± 0.01
	0.11 ± 0.01	0.11 ± 0.01	0.11 ± 0.01	0.09 ± 0.01

The highest seedling emergence (77.5–87.5%) was noted in seeds with an MC of 48.5–21.6%. However, these seeds did not produce seedlings after cryostorage (Fig. 1b). Although desiccation did not affect the survival or regrowth of explants in the in vitro culture, the embryonic axes excised from seeds with an MC of 21.6–48.5% did not survive cryostorage (Fig. 1c, d).

The TTC assay demonstrated high metabolic competence in both non-stored and cryostored seeds. However, there was a decline in metabolic activity at MC levels above 9.7%. Additionally, a greater difference in respiratory activity was observed between seeds with MCs of 48.5% and 41.4% that were stored and not stored in LN (Fig. 1E). However, despite high metabolic competence, the viability of seeds, as measured in germination and seedling emergence tests as well as embryonic axes in vitro survival and regrowth, appeared to be residual in cryostored seeds at high MCs (48.5–21.6%). These results corroborate the findings of a previous study on *A. platanoides*, which showed the highest survival rates in embryonic axes excised from seeds with an MC of less than 20% and stored in LN for 24 h^47^. Indeed, seed viability decreases when MC is excessively high due to the formation of ice crystals in membranes serving as nucleation sites in cryostored tissues^48,49^. Ice crystals cause membrane injuries, leading to electrolyte leakage^48,50^. In the current study, the measurement of electrolyte leakage demonstrated that seed desiccation caused membrane damage and increased electrolyte outflow. In cryostored seeds, electrolyte leakage was intensified at all MC levels, and the most pronounced increase was observed at the three highest MC levels, where electrolyte leakage was 8.7 to 4.3 times greater than in seeds subjected to desiccation alone (Fig. 1f).

### Changes in the reactive oxygen species content of embryonic axes and cotyledons excised from seeds with varying moisture content levels before and after cryostorage

**Fig. 2:**
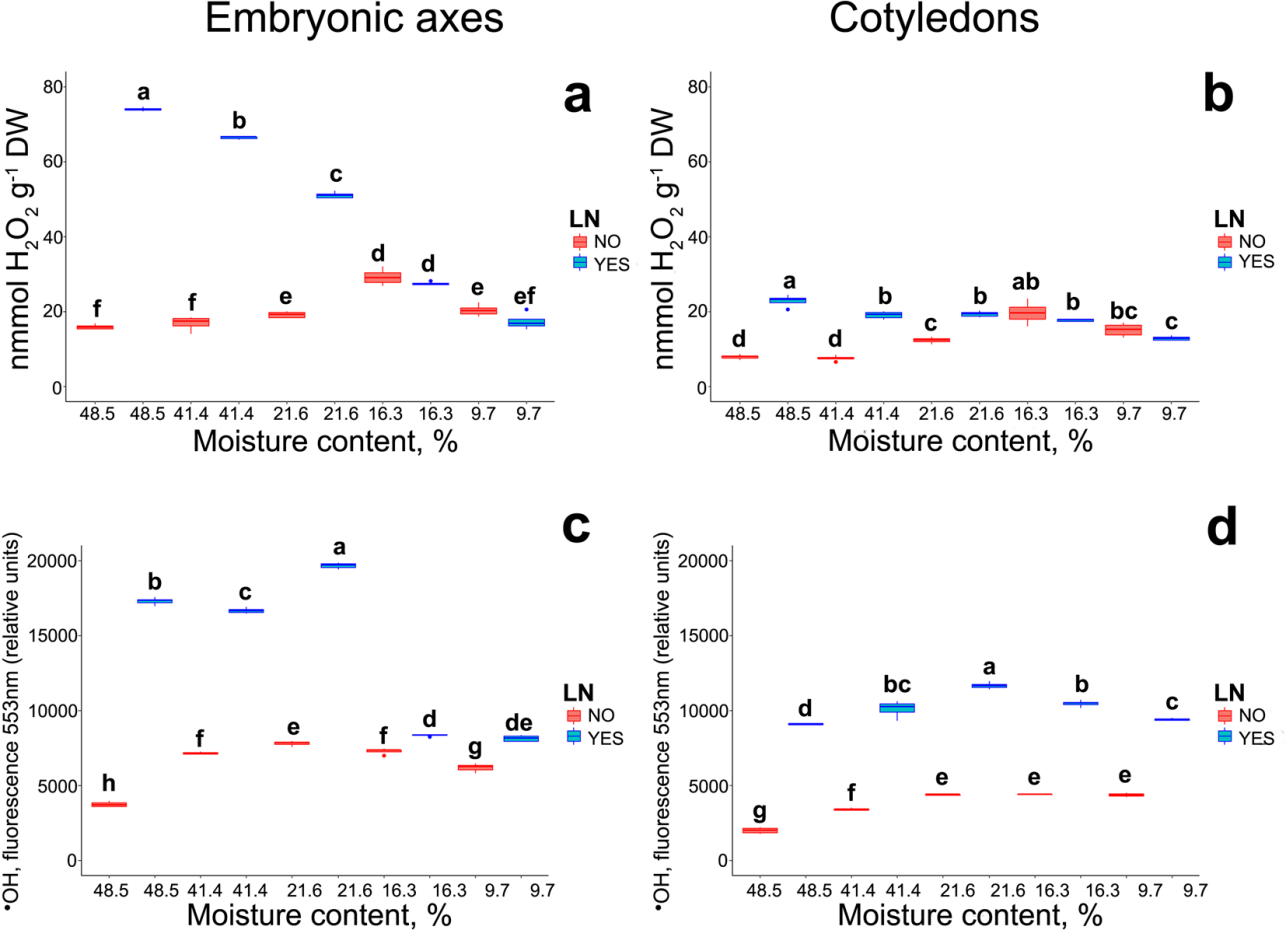
Effect of storage at different levels of MC in LN for 3 months on the level hydrogen peroxide in embryonic axes (**a**) or cotyledons (**b**), hydroxyl radicals in embryonic axes (**c**) or cotyledons (**d**) of *Acer platanoides* L. Statistical analysis was conducted using non-parametric Kruskal–Wallis test. Values marked with the same letter are not significantly different at *p* < 0.05. Lines denote the median, boxes the 25th to 75th percentile, while whiskers are the 5th to 95th percentiles.

Studies on seed physiology have shown that ROS play crucial roles in cell signaling that underpins seed germination and the breaking of seed dormancy, as well as in protection against pathogens^20,51^. On the other hand, it has been reported that ROS accumulation typically accompanies membrane damage, resulting in lipid peroxidation and programmed cell death (PCD)^48,52,53^. Notably, lipid oxidation products can induce DNA damage and interrupt the normal functioning of several cellular systems^54^. Consequently, the subsequent step of the analysis focused on determining the changes in ROS levels. After cryostorage, hydrogen peroxide (H_2_O_2_) content was higher in embryonic axes and cotyledons excised from seeds with an MC of 48.5–21.6%, and the absolute values were 2.5-3 times higher in embryonic axes than in cotyledons. No significant differences were observed in embryonic axes or cotyledons excised from seeds with a lower MC (Fig. 2a, b). Hydroxyl radical (^•^OH) levels were higher in both the embryonic axes and cotyledons excised from cryopreserved seeds with all MC levels. However, the highest absolute values were noted in the embryonic axes excised from cryostored seeds with an MC of 21.6–48.5% (Fig. 2c, d).

### DNA strand breaks and alterations in DNA modifications representing damage markers and epigenetic marks

**Fig. 3:**
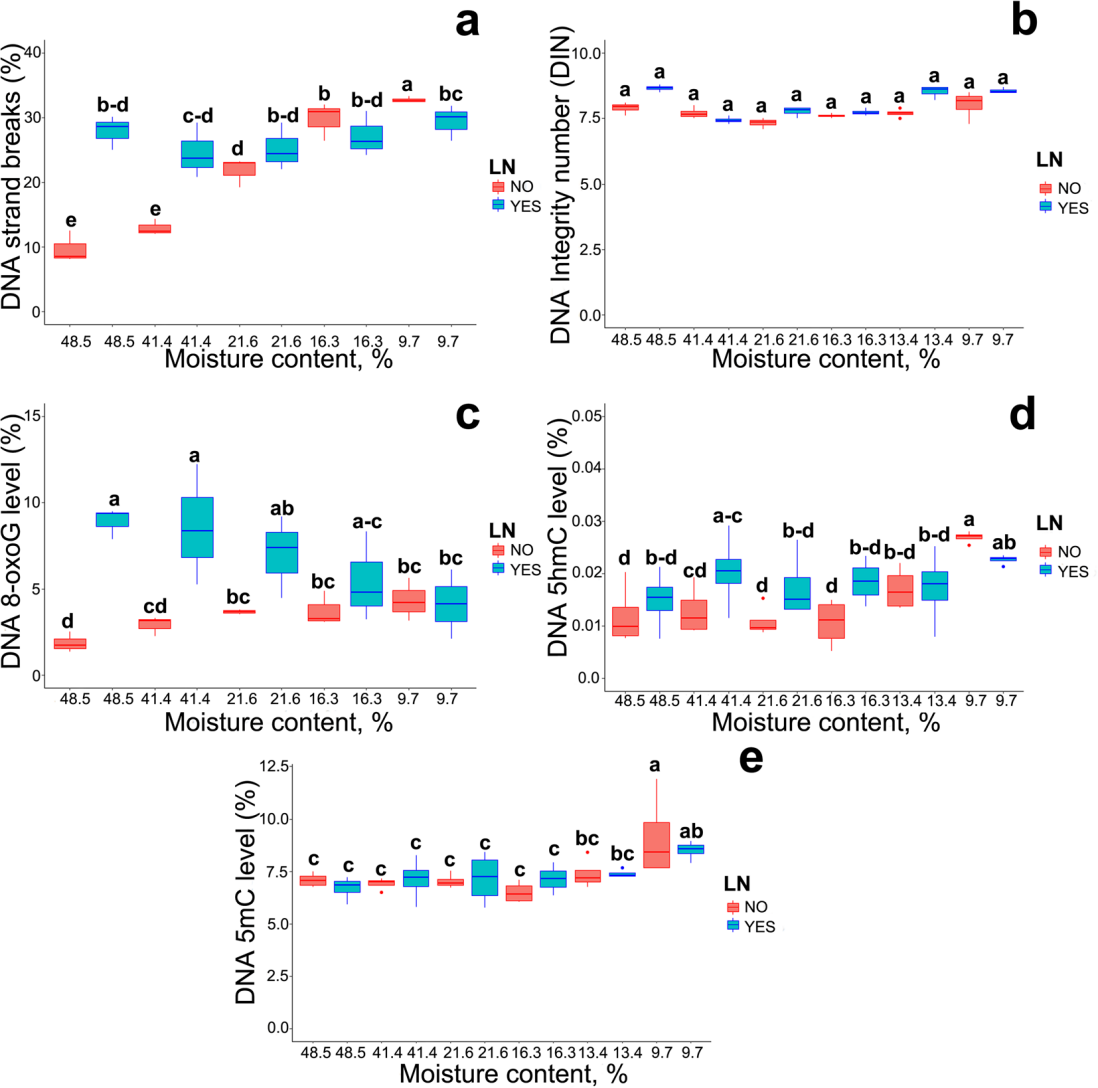
Effect of storage at different levels of MC in LN for 3 months on DNA strand breaks (% DNA in the tail) (a), DNA integrity (measured by DIN) (b), the relative global level of 8-oxo-7,8-dihydroguanine (c), 5-methylcytosine (d), 5-hydroxymethylcytosine (e), of *Acer platanoides* L. seeds. Statistical analysis was conducted using the LM and Duncan test for all measured parameters except DNA integrity (measured by DIN) (b) where the non-parametric Kruskal–Wallis test was conducted. Values marked with the same letter are not significantly different at *p* < 0.05. Lines denote the median, boxes the 25th to 75th percentile, while whiskers are the 5th to 95th percentiles.

The number of DNA strand breaks (SB) measured by the comet assay increased with the progression of desiccation, from around 10% of tail DNA in axes with an MC of 48.5% to around 30% of tail DNA in axes with an MC of 9.7%. In contrast, in embryonic axes excised from cryostored seeds, the percentage of DNA strand breaks remained unchanged at around 30% for all MC levels. Significant differences in the number of DNA strand breaks between non-stored and cryostored seeds were observed in seeds with the highest MC of 48.5% and 41.4% and seeds with an MC of 9.7% (Fig. 3a).

Automated capillary electrophoresis was used to assess DNA integrity (DNA integrity number, DIN) in comparison to the results of DNA strand breaks (SBs) obtained through the comet assay. Previous studies have shown no changes in the DIN of tumor tissues that have been cryopreserved for more than 10 years^55,56^. However, no such data are available for cryopreserved plant tissues. Here, the determination of DNA integrity by automated electrophoresis showed no differences between the tested samples, regardless of their MC or LN exposure (Fig. 3b).

Desiccation and cryostorage lead to oxidative stress. Thus, 8-oxoguanine (8-oxoG) levels in DNA were evaluated using an FPG-modified comet assay. Due to its lowest redox potential (-3.0 V), 8-oxoG is the most prevalent oxidized form of nucleobases, generated by reacting with oxygen at the C8 position, of which the double bond is directly attacked by ^•^OH, the most aggressive ROS^57^. However, recent reports indicate that carbonate radicals (CO_3_^**•**─^) specifically generate C8 or C5-oxidised G, but do not cause direct DNA SBs, whereas ^•^OH activity leads mainly to the generation of sugar-phosphate oxidation products^41^. The current research showed that 8-oxoG concentration increased significantly in embryonic axes desiccated to an MC of 21.6%, and it remained at a similar level during further desiccation with a maximum change of 2.47% compared to fresh control seeds. The cryostorage of seeds with the three highest MC levels increased 8-oxoG content to a maximum of 8.91% (Fig. 3c).

DNA is a highly dynamic molecule that undergoes various chemical modifications that act as regulatory marks, with the most important epigenetic modification – 5mC. Hydroxylated 5-methylcytosine (5hmC) is considered as a second epigenetic mark in eukaryotes. However, contrary to 5mC and 8-oxoG, the origin of 5hmC in the plant genome is still elusive^44,58,59^. Here, the measurements of the percentage of 5mC and 5hmC revealed a significant increase only at the lowest level of MC for both cryostored and non-cryostored seeds (Fig. 3d, e).

### Correlation analysis of seed viability, moisture content, reactive oxygen species levels, DNA strand breaks, and the appearance of DNA modifications in seeds before and after cryostorage 

**Fig. 4:**
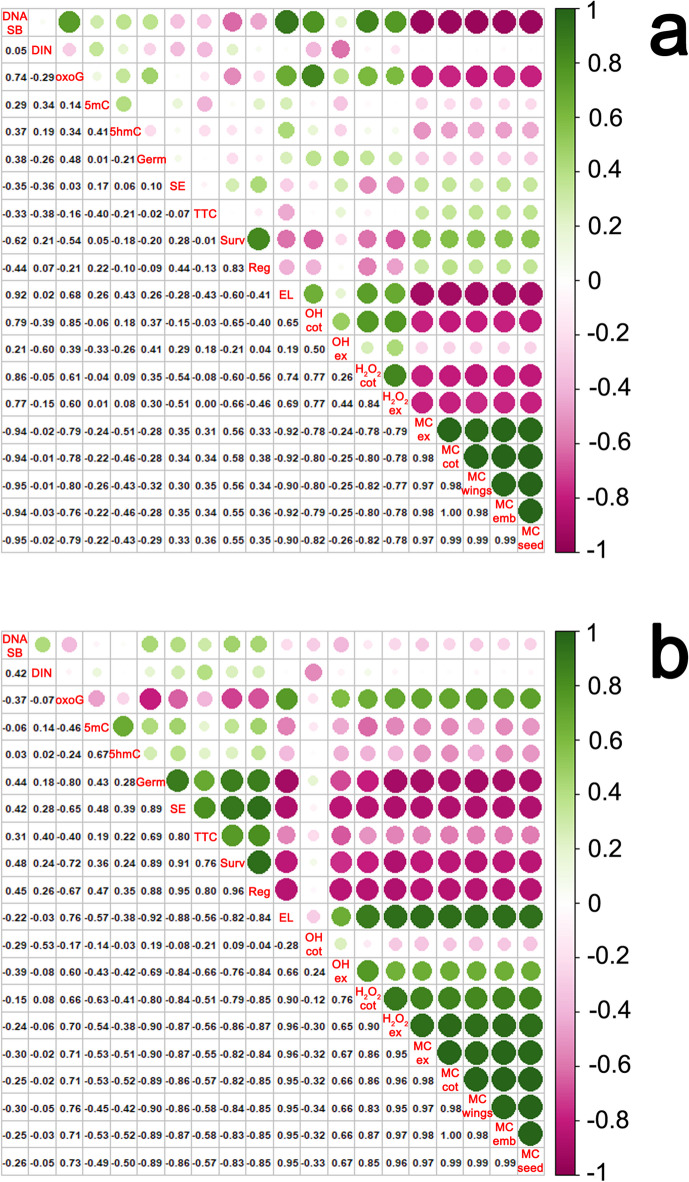
The Spearman correlation coefficient for desiccated seeds (a), or cryopreserved seeds (b). The Spearman correlation coefficient was analyzed between the means of DNA strand breaks (DNA SB), DNA integrity number (DIN), the relative global level of 8-oxo-7,8-dihydroguanine (oxoG), 5-methylcytosine (5mC) and 5-hydroxymethylcytosine (5hmC), germination (Germ), seedling emergence (SE), viability measured by TTC (TTC), in vitro survival (Surv), in vitro regrowth (Reg), electrolyte leakage (EL), hydrogen peroxide in cotyledons (OH cot) or embryonic axes (OH ex), hydrogen peroxide in cotyledons (H_2_O_2_ cot) or embryonic axes (H_2_O_2_ ex), moisture content of embryonic axes (MC ex), moisture content of cotyledons (MC cot), moisture content of wings (MC wings) moisture content of embryos (MC emb) moisture content of seeds (MC seed). The size of the circles represents the level of correlation, bigger circles indicate that a given trait correlates at a higher level. The green color indicates positive correlations and pink indicates negative correlations.

Desiccation and the MC of seeds were bound by a significant negative correlation with ROS, electrolyte leakage, DNA strand breaks, and 8-oxoG, while ^•^OH and H_2_O_2_ levels were positively correlated with 8-oxoG and DNA strand breaks (Fig. 4a). The MC of cryostored seeds was highly positively correlated with ROS, electrolyte leakage, and 8-oxoG, and negatively correlated with all parameters describing the viability of seeds and embryonic axes. The correlation analysis also revealed a highly significant negative correlation between electrolyte leakage and the viability of cryostored seeds (Fig. 4b). This observation corroborated the results of previous studies, which found a link between membrane integrity and germination capacity in orthodox seeds^54,60,61^. The relationship between seed viability and the tested modified bases was different. These factors were bound by a stronger correlation in cryostored seeds than in desiccated seeds. The viability was negatively correlated with the amount of 8-oxoG and positively correlated with epigenetic marks. Moreover, in cryostored seeds, ROS levels were highly negatively correlated with viability parameters, while in desiccated seeds, these correlations were unequivocal.

## Principal component analysis (PCA)

**Fig. 5:**
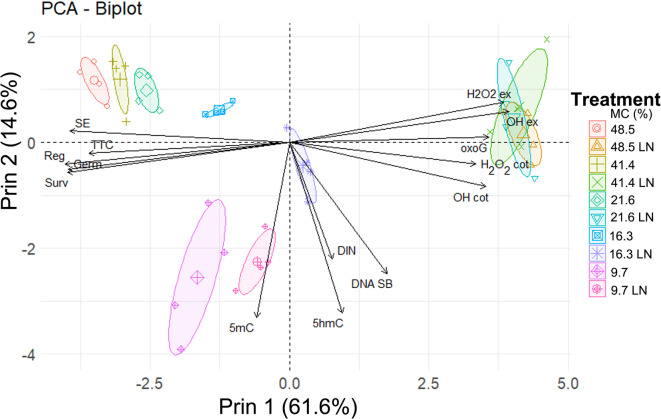
Results of principal component analysis (PCA) for combined data of desiccated and cryopreserved seeds. PCA was applied on the correlations of DNA strand breaks (DNA SB), DNA integrity number (DIN), the relative global level of (C), 8-oxo-7,8-dihydroguanine (oxoG), 5-methylcytosine (5mC) and 5-hydroxymethylcytosine (5hmC), germination (Germ), seedling emergence (SE), viability measured by TTC (TTC), in vitro survival (Surv), in vitro regrowth (Reg), electrolyte leakage (EL), hydrogen peroxide in cotyledons (OH cot) or embryonic axes (OH ex), hydrogen peroxide in cotyledons (H_2_O_2_ cot) or embryonic axes (H_2_O_2_ ex). Ellipse confidence level = 0.95.

Based on PCA, the results obtained for all experimental treatments were clustered into four groups, clearly separating cryostored and non-cryostored seeds based on their MC (Fig. 5). Groups 1, 2 and 3/4 were primarily distinguished based on the first principal component (Prin1), while groups 3 and 4 were distinguished based on Prin2. A strong negative relationship was found between viability parameters measured with all methods (coordinates in the range of -0.868 and − 0.957) and oxidative stress parameters ^•^OH, H_2_O_2_, and 8-oxoG (coordinates in the range of 0.807 and 0.951). This relationship was reflected by the opposite coordinates of these two groups of parameters and the high loadings of these variables in Prin1 (Supplementary Table S1a-c). Parameters 5mC, 5hmC DNA SB, and DIN had high loadings in the second principal component (Prin2).

## Discussion

In the current study, the MC, ROS levels, membrane integrity, metabolic competence and viability of cryostored orthodox seeds were analyzed to determine their relationships with the genotoxic effects of the freeze-thaw cycle. The study demonstrated that orthodox seeds are highly resistant to genotoxic stress and retain high viability when cryostored after desiccation to a low MC. However, immediate DNA damage (measured by the comet assay), along with a release of ROS and a loss of viability, was observed when the analyzed seeds were cryostored for 3 months at non-optimal, high MC. (Fig. 1f).

While seed germination implies an expansion process restarting the growth of the embryonic axis completed by radicle emergence through the seed covering layers, cotyledons provide various nutrients (fatty acids, carbohydrates, mineral nutrients, etc.) for this process^62,–64^. In this study, ROS components (^•^OH and H_2_O_2_) were measured separately in embryonic axes and cotyledons because oxidative stress does not occur uniformly throughout seeds^52,65^. This is expected as respiration rates decline more rapidly in cotyledon tissue than in embryonic axis tissue^66^. In seeds with a high MC, ^•^OH and H_2_O_2_ levels increased significantly in the embryonic axes after cryostorage. In contrast, a smaller increase in ROS levels was noted in the embryonic axes of seeds that were subjected to desiccation alone or were cryostored at a lower MC. Notably, the levels of both ROS components were similar in seeds with a low MC, regardless of whether they were cryostored. This indicates that cryostorage at a low MC does not intensify oxidative stress in the embryonic axes. It should also be noted that embryonic axes and cotyledons differed in the absolute content of •OH and H_2_O_2_, which points to the heterogeneity of the examined tissues. Statistical analyses revealed a much stronger negative correlation between ROS and the viability of cryostored seeds than seeds that were subjected to desiccation only (Fig. 4). In other studies, ROS levels were also high in cryostored seeds and other plant tissues^48^. Moreover, although the production of ROS is unavoidable in aerobic organisms, orthodox seeds have developed the ability to reduce the damage caused by water withdrawal, even when desiccated. This is achieved through the accumulation of late embryogenesis abundant proteins, heat shock proteins, and sugars, as well as an efficient system of antioxidant proteins and small molecule antioxidants that protect cells against a redox imbalance. Therefore, despite ROS accumulation being noted in desiccated seeds and seeds that were cryostored after desiccation, the seeds from both groups remained viable, proving their efficient antioxidant capacity characteristics of orthodox seeds. After characterizing the differences between desiccated and cryostored seeds varying in viability in terms of MC and the associated structural (membrane integrity) and metabolic (ROS accumulation) changes, the next research question was formulated: Does cryostorage induce immediate genotoxicity in seeds? Although ROS are generally recognized as a threat to DNA stability, leading to oxidation of sugar residues, DNA SBs, base modification, and protein-DNA crosslinking^22,67^the question of whether cryostorage induces immediate signs of ROS-related genotoxicity has remained unanswered. First, this problem was examined by analyzing the effect of a decrease in MC on the DNA integrity of orthodox seeds. Desiccation of *A. platanoides* seeds induced a genotoxic effect by increasing the number of DNA SBs in the embryonic axes by up to 30%, which is indicative of moderate damage^68^. Similar results were reported in recalcitrant embryonic axes of *Acer pseudoplatanus* L. subjected to desiccation^41^. However, orthodox seeds were more resistant to DNA damage than recalcitrant seeds, in which increased DNA damage was linked to reduced viability^69^. Thus, although DNA damage accumulation is observed during desiccation (Fig. 3), seeds do not lose their viability, and damaged DNA can be repaired during seed imbibition, which minimizes the mutational and inhibitory effects of accumulated DNA damage^69^. It has been proposed that the desiccation tolerance of orthodox seeds depends on their ability to repair genomic DNA when a desiccated embryo is rehydrated^70,71^. Next, the analysis of DNA SBs was repeated for cryostored seeds. Importantly, this is the first study to demonstrate that in seeds with a high MC, the percentage of DNA SBs is 2–3 times higher in cryostored than in non-cryostored seeds immediately after thawing. The detrimental effects of cryostorage on seeds with a high MC have been previously described^6,8^however, the notion that ice crystals cause mechanical damage to DNA has been challenged, despite the fact that ice crystals were detected in the nucleus^72,73^. To date, there has been no evidence of instant DNA fragmentation in plant cells immediately after cryostorage. Therefore, it can be assumed that the observed DNA damage was most likely caused by a redox imbalance and degradation of cellular structures, including the plasma membrane^74,75^ observed in the current study through electrolyte leakage analysis. Notably, the results of research conducted on human peripheral blood mononuclear cells (PBMCs) suggest that the freeze-thaw cycle is unlikely to exert a negative influence on the activity of DNA repair enzymes. Instead, it has been proposed that the observed increase in % tail DNA after thawing may be associated with impaired membrane integrity of thawed cells, leading to the release of nucleases from cell organelles^27^, and contributing to DNA damage. Intracellular water expands upon freezing, causing mechanical damage to organelles or membranes, which leads to cytolysis^75^. Thus, while the number of DNA SBs was high after cryostorage of seeds with a high MC and after desiccation, viability was low only in seeds with high electrolyte leakage, which could be attributed to observed membrane damage, possibly leading to impaired DNA repair by nuclease release, and cytolysis.

In the current study, DNA fragmentation was not detected as the DIN was similar in all tested samples (Fig. 3b). In previous research, standard agarose electrophoresis revealed no signs of DNA fragmentation in cryostored pollen. However, in comparison to those methods, the comet assay is a highly sensitive tool for detecting DNA damage, and its dynamic range has been estimated to be several hundred to several thousand breaks, which supports the detection of fine DNA damage^41^. Research on human PBMCs revealed that 25% of tail DNA represents 1.17 lesions per 10^6^ bp^76^which explains why DNA SBs were identified by the comet assay but not by automated capillary electrophoresis. Consequently, results obtained by automated capillary electrophoresis may be misleading. Moreover, previous studies have shown that small intracellular ice crystals in cryopreserved recalcitrant embryonic axes of *Acer saccharinum* L. provoked PCD in plants through respiratory burst, leading to autophagic decomposition of cell components^73^. Indeed, ROS-induced PCD is regarded as one of the primary factors influencing changes in the viability of cryopreserved plant materials^48^. In cryostored *Peonia emodii* seeds, higher ROS levels and higher activity of caspase-3-like protease, which contribute to PCD, were observed in seeds with a higher MC than in desiccated seeds^48,77^. However, significant DNA fragmentation, a late indicator of cell apoptosis, was not detected in the cited research or in the current study. Therefore, the comet assay appears to be a more effective technique for detecting early cryostorage-induced DNA damage in plant cells than traditional or automated electrophoresis.

In the presented research, the relative level of 8-oxoG increased during desiccation, although the percentage of 8-oxoG did not exceed 5% at any stage. In another study, the percentage of 8-oxoG was higher in recalcitrant embryonic axes, peaking at 17% in seeds with an MC of 19.3%^41^. The content of 8-oxoG also increased significantly to 10% in recalcitrant embryonic axes from non-dormant *Acer saccharinum* L. seeds that were desiccated and conventionally stored for 6 months^42^. Thus, the distinction between orthodox and recalcitrant seeds is also evident at the DNA oxidative damage level, as the 8-oxoG appears to a lesser extent during the desiccation of orthodox seeds (Fig. 3c), likely due to an efficient antioxidative system. However, the relative content of 8-oxoG measured immediately after thawing was higher in the embryonic axes from cryopreserved seeds with a higher MC than in their non-cryostored counterparts. The chromatographic (HPLC) method has been previously used to measure 8-oxoG concentration in currants cryopreserved at a temperature of − 20 and − 196°C^78^. However, the measurements were conducted after three regrowing periods of 6 to 8 weeks following cryostorage. During that time, damaged DNA nucleobases might have been repaired, which could explain why 8-oxoG levels did not increase. Therefore, to the best of the authors’ knowledge, this is the first study to demonstrate the presence of 8-oxoG in cryopreserved plant tissues.

Both 5mC and 5hmC were traced to compare epigenetic stability in orthodox seeds cryopreserved at varied MC levels. Desiccation induced oxidative stress, led to a redox imbalance, and exerted genotoxic effects, nevertheless, changes in global 5mC levels were detected only after desiccation to the lowest MC of 9.7% (Fig. 3d). Previous research on orthodox *A. platanoides* seeds characterized by high germination capacity and seedling emergence after gradual desiccation showed no significant changes relative to the control^79^. Similarly, the level of 5mC in *Pyrus communis* L. seeds did not change during desiccation to around 5% MC. However, additional desiccation up to 2% MC led to an increase in 5mC levels, but did not induce changes in germination or seedling emergence^10,80^. In intermediate *Populus nigra* L. seeds, desiccation from 12.5 to 14.5% to 7.6–7.7% MC increased DNA methylation, but did not lead to changes in seed germination^45^. Importantly, seed storage in LN did not change global 5mC levels in embryonic axes, measured immediately after thawing. Similarly, no significant change in global 5mC levels was observed after the cryopreservation of recalcitrant *Quercus robur* L. plumules^16^. In the authors’ previous study, desiccation-dependent changes were noted in 5hmC levels and were attributed to oxidation^44^. Indeed, ROS may directly affect methylation through their ability to oxidize 5mC^59,81^. However, in the current study, no increase in 5hmC levels was observed in the embryonic axes of orthodox seeds, except in seeds desiccated to the lowest moisture content. This finding contrasts with the observations made in recalcitrant embryonic axes, where a desiccation-dependent increase in 5hmC was detected^44^, proving once again the difference in resistance to desiccation between orthodox and recalcitrant seeds. Importantly, no difference in the amount of epigenetic marks was detected between cryostored and non-cryostored seeds. It seems that an efficient antioxidation system in the embryonic axes of *A. platanoides* ^82^ possibly contributes to epigenetic stability in seeds cryostored at different MC and storage in LN does not lead to epigenetic damage, at least when measured immediately after thawing.

## Conclusion

Cryopreservation is a method that allows for long-term preservation of valuable biological material. To ensure that this method is safe and preserves the integrity of biological species, it is essential to understand and monitor its effects on (epi)genetic information, and to select appropriate cryostorage procedures that do not compromise (epi)genetic integrity. This is the first study to demonstrate the genotoxic impact of LN storage on seeds at different MC analyzed immediately after the freeze-thaw cycle. The increase in the percentage of DNA SBs and 8-oxoG levels was associated with the high MC of seeds whose viability was compromised during cryostorage (Fig. 4). Moreover, a highly negative relationship between viability and oxidative stress-related parameters (ROS and 8-oxoG) was observed in all experimental treatments (Fig. 4). Importantly, multivariate analysis (PCA) clearly separated seeds subjected to storage in LN at high MC proving that ROS and 8-oxoG had the strongest negative impact on those seed viability among all tested parameters. Moreover, the relationship between ROS milieu and 8-oxoG was much stronger than with DNA SB, showing that 8-oxoG, a potentially mutagenic lesion, is a primary DNA damage product of oxidative stress resulting from the freeze-thaw cycle. Thus, the current results underline the importance of proper protocols and techniques for cryopreservation as a method for the conservation of true-to-type germplasm for long periods. It has also been proved that the comet assay is a valid and sensitive tool for detecting fine instant and potentially mutagenic DNA damage in cryostored seeds.

## Materials and methods

### Plant material and treatments

Winged seeds (samaras) of *Acer platanoides* L. (Norway maple) were collected from trees in the provenances in Kórnik, Poznań County, west-central Poland (52°14′42″ N, 17°5′55″ E). Samaras are a type of fruit characterized by a papery, flattened pericarp that forms wings encasing the seeds, which are flat nutlets. Samaras are dispersed by anemochory^83^. The water content (WC) of seeds was calculated on a dry weight basis and expressed as g H_2_O· g^-1^ dry mass (g·g^-1^), as well as on a fresh weight basis as a percentage of moisture content (MC), using a previously described formula^80^. The MC of freshly harvested seeds was 46.7%. To reduce MC, seeds were dried on a laboratory bench at 20 °C (Table 1). The average MC of samaras, seed coats, cotyledons, and embryonic axes was assessed by drying each part at 103 °C ± 2 °C for 24 h in four replications of five seeds per replicate. The MC and WC of samaras, seeds, cotyledons, and embryonic axes are presented in Table 1, and the MC of seeds is shown in the figures.

## Exposure to LN

Before cooling in LN, the wings of samaras were cut off at 2/3 of their length. The samaras were then placed into CryoFlex polyethylene bags (10 cm long and 5 cm wide, sealed at both ends). The seeds were cooled by direct immersion in LN, and they were stored in LN for three months. After LN storage, the bags containing the seeds were rewarmed in a 40 °C water bath for 10 min. Fresh or desiccated seeds were used as the control for cryostorage experiments.

## Stratification, germination, and seedling emergence tests

After desiccation, dormant *A. platanoides* seeds (inside samaras) had to be stratified before germination and seedling emergence tests. The stratification, germination and seedling emergence tests were performed as previously described^79^. In brief, the seeds were mixed with the substrate (1:3, v/v) and placed in plastic bottles until the first germinated seeds (with 2-3-mm-long radicals) were counted. Germination was a visible indicator that seeds had been released from dormancy. The plastic bottles containing seeds were monitored for fungal infections throughout the stratification treatment. Seeds were stratified for 12–13 weeks at 3 °C until more than 5% of the seeds had developed visible radicals. Stratified seeds were germinated (in 4 replicates of 50 seeds each) in darkness, in the same mixture of sand and peat that was used in stratification experiments. To ensure the optimal thermal conditions for germination, the seeds were exposed to alternating temperatures of 3 °C/20°C (16 h/8 h per day, respectively). A seed with a 3-mm-long radicle was considered germinated. Substrate identical to that used for seed stratification and germinatThe boxes with the seedlings were subjected ion was used in seedling emergence tests. Stratified seeds were sown in plastic boxes (in 4 replicates of 50 seeds each) to a depth of 1 cm and covered with a layer of the substrate. The boxes were covered with a transparent lid allowing light to pass through. The seedling emergence test was conducted under the same thermal conditions as the germination assay (3 °C/20°C, 16 h/8 h per day) until the seedlings achieved a height of approximately 20–30 mm. to a16 h/8 h photoperiod (60 µmolm^-2^ s^-1^) in a climate chamber set to a temperature of 20 °C. The emergence test lasted 4 to 5 weeks.

### Viability assessment by respiratory activity

The viability of desiccated and cryopreserved seeds was determined by the tetrazolium chloride (TTC) assay, as previously described^46^ Four biological replicates of thirty seeds each were stained in 1% 2,3,5-triphenyltetrazolium chloride (TTC) solution. The embryonic axes that remained green were considered dead, while those stained pink to red were classified as metabolically (respiratory) active and alive.

An in vitro regrowth assay was performed as previously described^46^using four biological replicates of 15–20 embryonic axes each. The survival of embryonic axes was assessed after four weeks of in vitro culture. The embryonic axes were considered alive (survived) when they developed shoots or roots, or increased in size. After eight weeks, the regrowth of the embryonic axes was assessed, and only those that had developed shoots or shoots with roots were regarded as regenerated.

### Electrolyte leakage

Seeds (4 replicates of 10 seeds each) were placed in 10 mL of deionized water. The conductivity of the solutions was measured after 24 h of incubation at room temperature using a conductivity meter (Mettler Toledo InLab 730). The results were expressed in µS per 1 g of the sample on a dry weight (DW) basis^42^.

### Oxidative stress markers in seeds

#### Determination of hydrogen peroxide (H_2_O_2_) levels

Samples composed of 10 embryonic axes or 5 cotyledons were analysed in four replications. Embryonic axes or cotyledons were homogenised in 5% trichloroacetic acid (TCA), as previously described^42^. The homogenate was centrifuged for 20 min at 4 °C at 2600xg. The reaction mixture contained 0.5 mL of the extract, 1.5 mL of 50% TCA, 0.4 mL of 10 mM ferric ammonium sulphate, and 0.2 mL of 2.5 M potassium thiocyanate. The control contained 0.5 m of 5% TCA instead of the extract. Absorbance was measured spectrophotometrically (Shimadzu UV-2501PC) at 480 nm. The amount of µg H_2_O_2_ was read from the previously prepared standard curve.

### Determination of hydroxyl radical (^•^OH) production

Hydroxyl radical production was determined as previously described^42^by incubating 10 embryonic axes and five cotyledons from each desiccation treatment in 1.5 mL of K-phosphate buffer (20 mM, pH 6.0) containing 20 mM 2-deoxy-D-Rib (Sigma–Aldrich, Poland). The formation of malondialdehyde as the decomposition product was determined by combining 0.5 mL of the centrifuged incubation medium with 0.5 mL of 2-thiobarbituric acid (TBA) (10 g·l^− 1^ in 50 mM NaOH) and 0.5 mL of TCA (28 g·l^− 1^). The reaction product was heated in boiling water for exactly 10 min, cooled in tap water, clarified by centrifugation, and measured fluorometrically (excitation: 532 nm, emission: 553 nm) against reagent blanks.

### DNA isolation and measurement of DNA modifications

Total genomic DNA was extracted as previously described^44^. Five embryonic axes were used as replicates in each experiment, and each experiment was replicated four times. The embryonic axes were homogenized in LN, and DNA was extracted using a NucleoSpin Plant II Kit (Macherey-Nagel, Düren, Germany) according to the manufacturer’s instructions. DNA concentration and quality (A_260_/A_280_ = 1.8–1.9) were measured with a NanoQuant Plate M200 PRO (Tecan, Männedorf, Switzerland). DNA modifications were quantified calorimetrically using MethylFlash Global DNA Methylation (5-mC) and Hydroxymethylation (5-hmC) ELISA Easy Kits (Epigentec, Farmingdale, NY, USA) according to the manufacturer’s instructions. Two technical replicates of each biological replicate were measured. A total of 100 ng of DNA was used in 5mC and 5hmC measurements. DNA integrity was analyzed as previously described^41^ using a TapeStation 4200 automated electrophoresis platform (Agilent Technologies, Santa Clara, CA, USA) with Genomic DNA ScreenTape (sizing range: 200 to > 60,000 bp) according to the manufacturer’s instructions.

### The comet assay

DNA strand breaks in the embryonic axes isolated from *Acer* sp. seeds were measured according to a previously presented procedure^41^. In brief, three biological replicates of five axes each were used in the comet assay for each tested condition. The embryonic axes excised from seeds were placed in a 60 mm glass Petri dish, kept on ice, and vigorously chopped (up to 30 s) in 50 mM Sörensen buffer (pH 6.8) supplemented with Na_2_EDTA and DMSO to a final concentration of 0.5 mM and 0.5%, respectively. The nuclear suspension (100 µl) was then gently mixed with 1% low melting-point agarose (LMP; TopVison Low Melting Point Agarose; Thermo Fisher Scientific, Waltham, MA, USA) pre-warmed to 42 °C. Subsequently, 50 µl aliquots were placed on three microscope slides pre-coated with 1% normal melting point agarose for (1) the alkaline comet assay; (2) the enzyme-modified version of the comet assay (+ FPG; formamidopyrimidine DNA glycosylase); or (3) the buffer control for the enzyme-modified version of the comet assay (− FPG). The slides were left in a lysis buffer (2.5 M NaCl, 100 mM Na_2_EDTA, 1% Triton X-100, 10%DMSO, 10 mM Tris, pH 10) for 1 h at 4 °C. Slides for the enzyme-modified comet assay (−/+ FPG) were washed three times for 5 min with FPG buffer (50 mM Tris-HCL, 2 mM Na_2_EDTA, KCl 50 mm, pH 7.5). Next, 50 µL of FPG enzyme (diluted 1:3000; 0.6 U/mL) in an FPG reaction buffer supplemented with BSA (0.2 mg/mL) were added to the gel, and the slides were covered with parafilm. Only the FPG reaction buffer was added to the control slides (− FPG) to determine net incisions. The slides were incubated at 37 °C for 30 min in a humidified box. Next, the slides were rinsed two times with 0.4 M Tris HCl pH 7.5 and placed in a dish containing freshly prepared, cold alkaline solution (1 mM Na2EDTA,300 mM NaOH, pH > 13) at 4 °C for 40 min to allow DNA to unwind before electrophoresis (20 min at 1 V/cm). The slides were neutralized with 0.4 M Tris-HCl (pH 7.5) twice for 5 min, dehydrated in methanol for 10 min, and dried overnight at RT. The slides were stained with SybrGold (dilution: 1:10,000 in 1x PBS, Invitrogen, Waltham, MA, USA) for 1 h and washed in dd H_2_O for 10 min prior to imaging. Typically, 30 randomly selected comets in a total of three biological replicates were scored using a fluorescent microscope (Leica TCS SP5 II, Germany), and the number of DNA strand breaks (% tail DNA) was recorded using the Comet Assay IV analytical software (Perceptive Instruments, Haverhill, Suffolk, UK).

### Statistical analysis

R software (R Core Team 2020; https://www.r-project. org) was used for the statistical analyses and graphical visualization of data. The effect of desiccation on viability (as measured by the TTC staining assay), survival, regrowth, germination, and seedling emergence was evaluated separately using a generalized linear model (GLM) with binomial distribution.

The normality of data distribution was assessed using the Shapiro–Wilk test, and the homogeneity of variance was determined using Levene’s test. 5mC was normally distributed. The Lambert W function was successfully applied to determine DNA strand breaks and 8-oxoG levels in DNA. Significant differences between means were determined by two-way ANOVA, and pairwise comparisons of treatment means were performed using Duncan’s multiple range test at *p* ≤ 0.05.

The Kruskal-Wallis non-parametric test was used (*p* ≤ 0.05) to determine significant differences between groups of data with non-normal distribution or data with non-homogenous variance, including the amount of electrolyte leakage, hydroxyl radicals, superoxide anions, hydrogen peroxide, and DNA integrity (DIN).

Spearman’s correlation analysis was conducted to examine the relationship between germination, seedling emergence, in vitro regrowth, and viability (measured by the TTC assay) vs. hm5C, m5C, and 8-oxoG levels, DNA strand breaks, electrolyte leakage, hydroxyl radicals, and hydrogen peroxide levels in seeds with varying MC.

The correlation matrix was visualized with the R corrplot package. The dataset was transformed by z-score normalisation in the principal component analysis (PCA) using ggplot2, factoextra, and FactoMineR packages in R. Data were visualized using the R ggplot2 package.

## Electronic supplementary material

Below is the link to the electronic supplementary material.

Supplementary Material 1

## Data Availability

The datasets used and/or analyzed during the current study are available from the corresponding author upon reasonable request.
