# Supplementary material for: Impact of cryopreservation on DNA damage in Acer platanoides L. seeds evaluated by the comet assay
Source: Sci Rep. 2025 Jul 5;15:24081. doi: 10.1038/s41598-025-08476-3 (PMC12228746; doi:10.1038/s41598-025-08476-3)
Supplement: Supplementary file 1 — Supplementary Material 1 [file 41598_2025_8476_MOESM1_ESM.pdf]

Supplementary Table S1a. Quality of representation for variables on the factor map of PCA. Principal component analysis (PCA) results for combined data of desiccated and cryopreserved seeds. PCA was applied on the correlations of DNA strand breaks (DNA SB), DNA integrity number (DIN), the relative global level of (C), 8-oxo-7,8-dihydroguanine (oxoG), 5-methylcytosine (5mC) and 5-hydroxymethylcytosine (5hmC), germination (Germ), seedling emergence (SE), viability measured by TTC (TTC), in vitro survival (Surv), in vitro regrowth (Reg), electrolyte leakage (EL), hydrogen peroxide in cotyledons (OH cot) or embryonic axes (OH ex), hydrogen peroxide in cotyledons (H_2O_2 cot) or embryonic axes (H_2O_2 ex).

	**Dim.1**	**Dim.2**	**Dim.3**	**Dim.4**	**Dim.5**	**Dim.6**	**Dim.7**	**Dim.8**	**Dim.9**	**Dim.10**	**Dim.11**	**Dim.12**
DNA SB	0.178214	0.357619	0.369293	0.049949	0.001746	0.003031	0.001899	0.029286	0.000808	0.007364	0.000442	1.4E-05
DIN	0.035021	0.283521	0.000818	0.672053	6.42E-05	0.000143	0.000634	0.006471	4.9E-05	0.000914	0.000246	5.05E-06
8oxoG	0.745997	0.000528	0.006872	1.45E-05	0.000674	0.061395	0.181442	0.001074	0.001034	0.000195	0.000699	4.33E-05
m^5C	0.020605	0.63907	0.119667	0.019221	0.166724	0.01239	0.010679	0.011075	0.000344	0.000151	1.87E-05	1.66E-05
5-hmC	0.052356	0.607319	0.079365	0.039524	0.100623	0.106275	0.012818	0.000595	0.000234	0.000498	0.000274	7.26E-05
Germ	0.915813	0.015253	0.03664	0.012757	0.003146	6.04E-05	0.000872	0.002651	0.000604	4.95E-05	0.004009	0.002799
SE	0.903715	0.002785	0.000131	0.005685	0.011995	0.037455	0.000333	0.020817	0.008966	0.001928	1.79E-05	0.005975
TTC	0.753816	0.002602	0.117654	0.036266	0.005159	0.014654	0.003508	0.036668	0.020644	0.008549	7.4E-05	4.34E-05
Surv.	0.926169	0.019034	0.009852	3E-05	0.010812	0.013728	0.005975	0.002215	0.003309	0.00328	0.002311	5.9E-05
Reg.	0.942885	0.010029	0.004029	6.45E-06	0.001054	0.002173	0.012193	0.00111	0.000518	0.003669	0.020709	0.000337
OH cot	0.723796	0.040871	8.87E-05	0.004488	0.075434	0.130057	0.014975	0.001639	0.005659	0.000142	2.04E-05	0.001391
OH ex	0.905422	0.020086	0.011708	0.000229	0.00818	0.001523	0.000187	0.014295	0.030372	0.002485	0.003227	0.00179
H_2O_2 cot	0.65173	0.010023	0.260709	0.006396	0.011732	0.001976	0.007377	0.02495	5.19E-05	0.024633	0.000293	7.46E-05
H_2O_2 ex	0.863848	0.034027	0.00098	0.009905	0.029074	0.032465	0.002577	0.005625	0.001383	0.005715	0.010947	0.000242

Supplementary Table S1b. Coordinates of principal component analysis. Principal component analysis (PCA) results for combined data of desiccated and cryopreserved seeds. PCA was applied on the correlations of DNA strand breaks (DNA SB), DNA integrity number (DIN), the relative global level of (C), 8-oxo-7,8-dihydroguanine (oxoG), 5-methylcytosine (5mC) and 5-hydroxymethylcytosine (5hmC), germination (Germ), seedling emergence (SE), viability measured by TTC (TTC), in vitro survival (Surv), in vitro regrowth (Reg), electrolyte leakage (EL), hydrogen peroxide in cotyledons (OH cot) or embryonic axes (OH ex), hydrogen peroxide in cotyledons (H_2O_2 cot) or embryonic axes (H_2O_2 ex).

	**Dim.1**	**Dim.2**	**Dim.3**	**Dim.4**	**Dim.5**	**Dim.6**	**Dim.7**	**Dim.8**	**Dim.9**	**Dim.10**	**Dim.11**	**Dim.12**
DNA SB	0.422154	-0.59801	-0.60769	-0.22349	0.041787	-0.05506	0.043573	-0.17113	0.02843	-0.08581	0.021022	-0.00374
DIN	0.187139	-0.53247	-0.0286	0.819788	0.008012	-0.01198	0.025178	-0.08044	0.006999	0.030225	-0.0157	-0.00225
8oxoG	0.863711	0.022978	-0.0829	0.003807	-0.02596	0.247781	-0.42596	-0.03277	0.032152	0.013978	-0.02643	0.006581
m^5C	-0.14355	-0.79942	0.34593	-0.13864	0.408319	-0.11131	-0.10334	0.105236	-0.01855	-0.01231	-0.00433	-0.00408
5-hmC	0.228814	-0.77931	0.281717	-0.19881	-0.31721	0.325998	0.113216	0.024388	-0.01529	0.022317	0.016563	0.008522
Germ	-0.95698	-0.1235	-0.19141	-0.11295	0.056085	0.007771	-0.02952	-0.05149	-0.02458	0.007036	-0.06332	0.05291
SE	-0.95064	0.052771	-0.01144	-0.0754	0.10952	0.193533	-0.01824	-0.14428	-0.09469	0.043909	-0.00423	-0.0773
TTC	-0.86823	-0.05101	-0.34301	0.190436	-0.07183	0.121054	-0.05923	0.191488	-0.14368	-0.09246	0.008603	0.006584
Surv.	-0.96238	-0.13796	-0.09926	0.005477	-0.10398	-0.11716	-0.0773	0.047059	0.057521	0.05727	0.048068	-0.00768
Reg.	-0.97102	-0.10015	-0.06347	0.00254	-0.03246	-0.04661	-0.11042	-0.03331	0.022752	0.060571	0.143906	0.018349
OH cot	0.850762	-0.20217	0.00942	-0.06699	-0.27465	-0.36063	-0.12237	0.040479	-0.07523	-0.0119	0.004513	-0.0373
OH ex	0.951537	0.141725	0.108202	-0.01513	0.090441	-0.03903	0.013683	-0.11956	-0.17428	0.049848	0.05681	0.042303
H_2O_2 cot	0.807298	-0.10011	-0.5106	-0.07998	0.108316	0.044452	0.085889	0.157957	-0.0072	0.15695	-0.01712	-0.00864
H_2O_2 ex	0.929434	0.184465	-0.03131	0.099526	0.170511	0.180181	0.050761	0.074999	0.037192	-0.07559	0.10463	-0.01557

Supplementary Table S1c. The contributions (in percentage) of the variables to the principal components. Principal component analysis (PCA) results for combined data of desiccated and cryopreserved seeds. PCA was applied on the correlations of DNA strand breaks (DNA SB), DNA integrity number (DIN), the relative global level of (C), 8-oxo-7,8-dihydroguanine (oxoG), 5-methylcytosine (5mC) and 5-hydroxymethylcytosine (5hmC), germination (Germ), seedling emergence (SE), viability measured by TTC (TTC), in vitro survival (Surv), in vitro regrowth (Reg), electrolyte leakage (EL), hydrogen peroxide in cotyledons (OH cot) or embryonic axes (OH ex), hydrogen peroxide in cotyledons (H_2O_2 cot) or embryonic axes (H_2O_2 ex).

	**Dim.1**	**Dim.2**	**Dim.3**	**Dim.4**	**Dim.5**	**Dim.6**	**Dim.7**	**Dim.8**	**Dim.9**	**Dim.10**	**Dim.11**	**Dim.12**
DNA SB	2.067595	17.5066	36.28327	5.83164	0.409486	0.726335	0.743175	18.48061	1.092613	12.36091	1.020852	0.10855
DIN	0.406306	13.87928	0.080339	78.46283	0.015055	0.034377	0.248155	4.083538	0.066222	1.533502	0.569065	0.039229
8oxoG	8.654868	0.025847	0.675144	0.001692	0.158058	14.71165	71.0236	0.677852	1.397388	0.327998	1.613997	0.3367
m^5C	0.239059	31.28452	11.75741	2.244029	39.09904	2.96887	4.180127	6.988451	0.46502	0.254228	0.043307	0.129096
5-hmC	0.607421	29.73023	7.797621	4.614438	23.5973	25.46569	5.017443	0.37532	0.316118	0.836085	0.633734	0.564578
Germ	10.62503	0.746663	3.599862	1.489399	0.737665	0.014469	0.341204	1.672969	0.817009	0.083093	9.261994	21.76327
SE	10.48468	0.136326	0.012856	0.663764	2.812917	8.97504	0.130245	13.13613	12.11984	3.236495	0.041398	46.45122
TTC	8.745585	0.127379	11.55955	4.23406	1.209909	3.511456	1.373297	23.13852	27.9058	14.35024	0.170956	0.337028
Surv.	10.74519	0.931763	0.96794	0.003502	2.535437	3.289403	2.338803	1.397478	4.472665	5.505775	5.337453	0.458592
Reg.	10.93911	0.490967	0.395821	0.000753	0.247085	0.520592	4.772826	0.700279	0.699767	6.158769	47.83863	2.617402
OH cot	8.3973	2.000756	0.008718	0.523939	17.6902	31.16438	5.861605	1.034007	7.650311	0.237695	0.047055	10.81747
OH ex	10.50448	0.983275	1.150296	0.026736	1.918206	0.364939	0.073288	9.020776	41.05722	4.171212	7.455537	13.91192
H_2O_2 cot	7.561211	0.490644	25.61487	0.746766	2.751392	0.473485	2.887605	15.74457	0.070155	41.35116	0.67681	0.580013
H_2O_2 ex	10.02216	1.665745	0.096305	1.156457	6.818261	7.779312	1.008623	3.5495	1.869866	9.592835	25.28921	1.88493
